# Inhibition mechanism investigation of quercetagetin as a potential tyrosinase inhibitor

**DOI:** 10.3389/fchem.2024.1411801

**Published:** 2024-06-03

**Authors:** Faliang Liang

**Affiliations:** Pharmacy Department, Jiang Men Maternity and Child Healthcare Hospital, Jiangmen, China

**Keywords:** nature product, quercetagetin, tyrosinase, inhibition effects, inhibitor

## Abstract

Tyrosinase is one important rate limiting enzyme in melanin synthesis, directly affecting the melanin synthesis. Quercetagetin is one active ingredient from marigold. Thence, the inhibition effects of quercetagetin against tyrosinase were investigated. The results showed quercetagetin could inhibit tyrosinase activity with IC_50_ value of 0.19 ± 0.01 mM and the inhibition type was a reversible mixed-type. Results of fluorescence quenching showed quercetagetin could quench tyrosinase fluorescence in static process. CD and 3D fluorescence results showed the interaction of quercetagetin to tyrosinase could change tyrosinase conformation to inhibit activity. Moreover, docking revealed details of quercetagetin’s interactions with tyrosinase.

## 1 Introduction

**FIGURE 1:**
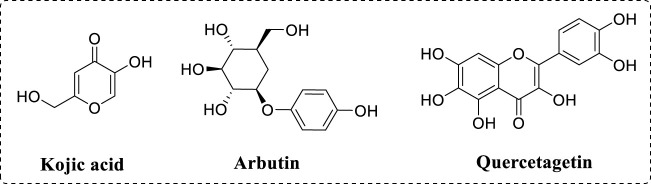
Chemical structure of kojic acid, arbutin and quercetagetin.

As we all know, various diseases affect people’s health (Zhang et al., 2020; Zhang X. et al., 2021; Shi et al., 2023; Zhou S. et al., 2022). As a therapeutic target, tyrosinase is one important metal enzyme containing two copper, which widely presents in the organism (Min et al., 2023; Fan et al., 2017). Tyrosinase has been confirmed to be involved in the synthesis of melanin (Li et al., 2023; Hassan et al., 2023; Li J. et al., 2021). The first reaction process is monophenolase activity, in which L-tyrosine is hydroxylated into L-dopa and the second reaction process is diphenolase activity, in which L-dopa is subsequently oxidized into dopaquinone (Djafarou et al., 2023; Lee et al., 2023; Zargaham et al., 2023). In the organism, melanin acts crucial roles to protect the skin from UV radiation (Lu et al., 2023a; Znajafi et al., 2023). But, excessive elevated melanin content leads to lots of pigmentation disorders, including age spots and melanoma (Broulier et al., 2023; De Barros et al., 2023; Xue et al., 2023). Inhibition of tyrosinase activity would reduce melanin generation, thence, the finding on novel tyrosinase inhibitors is attracting more attention due to their potential application in medicine and cosmetics fields (Wang and Mu, 2021). Although kojic acid and arbutin (Figure 1) are applied as tyrosinase inhibitors in the medical and industrial fields, they still have been found to lots of adverse side effects (Wang et al., 2021; Esma et al., 2023). Now, to find novel tyrosinase inhibitors is essential for treatment of melanin synthesis (Romagnoli et al., 2022; Nasab et al., 2023).

Natural products have been the vital sources for clinical drugs (Li Y. et al., 2021; Wu et al., 2021; Chen et al., 2022; Zang et al., 2022). Many natural products display widely biological activities, such as antioxidant (Zhang Y. et al., 2021; Tao et al., 2022; Tang et al., 2023), anti-tumor (Liu et al., 2022; Chen et al., 2023; Song et al., 2023), anti-inflammatory (Wang et al., 2020; Wang X. et al., 2022; Zhou Y. et al., 2022), and so on (Wang Y. et al., 2022; Qi et al., 2022; Ma et al., 2023). In particular, natural products show low toxicity (Shao et al., 2020; Jiang et al., 2021; Pei et al., 2021). Thence the development of natural products as tyrosinase inhibitors attracts much attention.

Quercetagetin (Figure 1) is one active ingredient from marigold and has a chemical structure of 3,3′,4′,5,6,7-hexahydroxyflavone (Bulut and Yilmaz, 2021; Wang X. et al., 2022). As one polyhydroxyphenol molecule with multiple hydrogen donor substituents, quercetagetin represents rich biological activities (Wu F. et al., 2023). For example, quercetagetin shows strong antioxidant activity and can effectively scavenge DPPH and ABTS (Fuentes et al., 2021). Quercetagetin also enhances the antioxidant enzymatic activities in broilers tissue by Nrf2/ARE signal pathway (Wu et al., 2022). In addition, quercetagetin displays immunomodulatory and anti-inflammatory to inhibit the release of macrophage-derived chemokine (Rufino et al., 2021). Besides, quercetagetin display other beneficial functions, including anti-virus and anti-diabetes (Seyedi et al., 2016). However, to our knowledge, the inhibition effects of quercetagetin against tyrosinase have not been reported yet.

Hence, in this study, we investigated the inhibition effects of quercetagetin against tyrosinase by the multispectral method, followed by the molecular docking.

## 2 Results and discussion

### 2.1 Inhibitory activity

**FIGURE 2:**
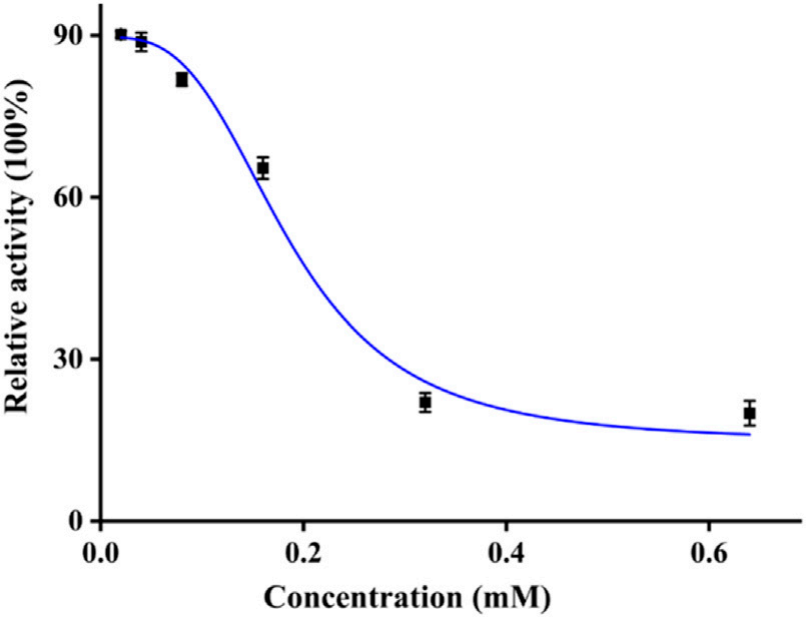
Tyrosinase inhibitory activity of quercetagetin.

The tyrosinase inhibitory activity of quercetagetin was examined on mushroom tyrosinase. The tyrosinase activity was measured under different concentrations of quercetagetin (Figure 2). It could be observed that tyrosinase relative activity gradually reduced with quercetagetin concentration (0–0.64 mM), meaning that quercetagetin could inhibit the tyrosinase activity with quercetagetin concentration. Its IC_50_ value was calculated to be 0.19 ± 0.01 mM, which was lower than that of kojic acid. This result showed that quercetagetin could be used as a natural tyrosinase inhibitor.

### 2.2 Kinetic study

**FIGURE 3:**
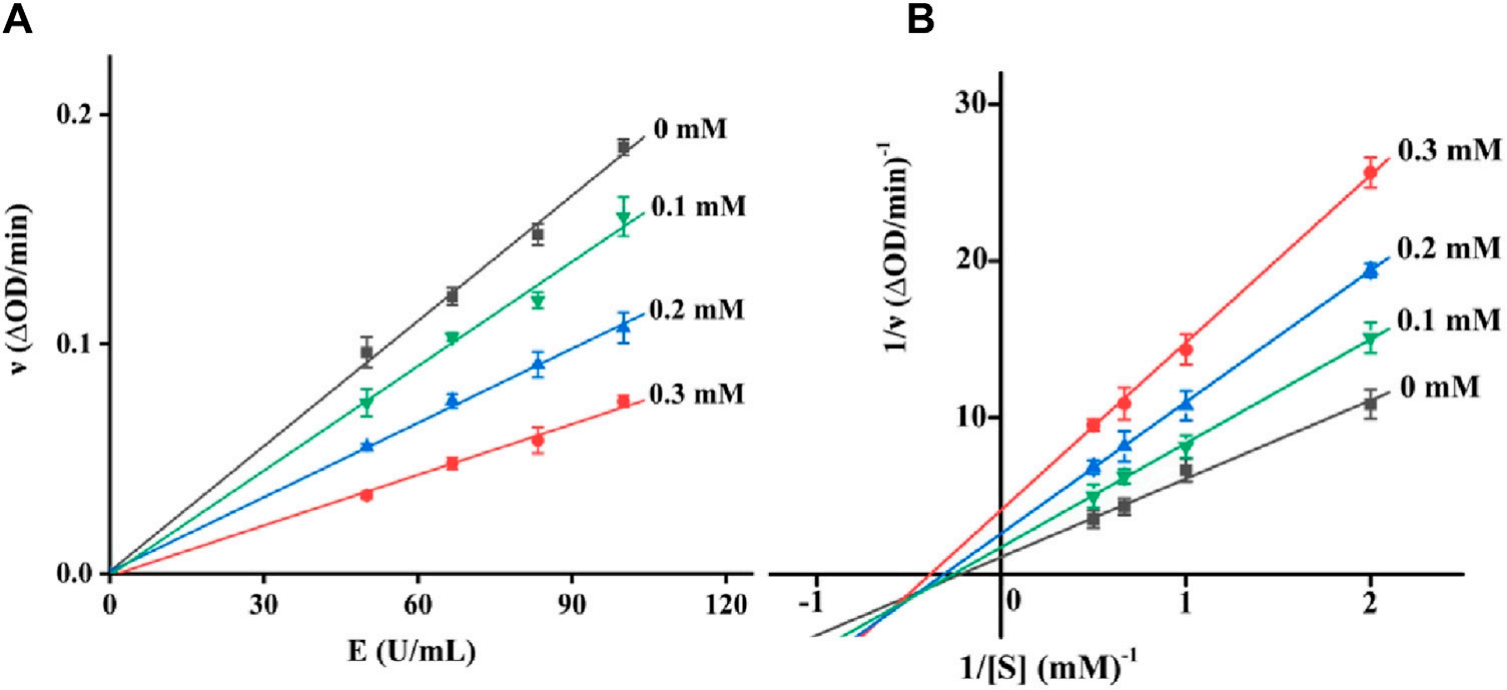
**(A)** Reversible assay of quercetagetin; **(B)** Lineweaver-Burk plots of quercetagetin.

There are two kinds of inhibitors, reversible and non-reversible inhibitors. For a reversible inhibitor, it can reduce the enzyme activity by the binding to enzyme, which can restore the enzyme activity through the remove of inhibitor. There are three reversible inhibitors, including competitive, non-competitive and mixed-type inhibitors. The inhibition type of quercetagetin on tyrosinase was subsequently investigated. With different concentration of quercetagetin and tyrosinase, the absorbance changes were measured (Figure 3A) and found that the lines of quercetagetin with different concentration passed origin and slopes reduced with quercetagetin concentration. The results suggested quercetagetin as a reversible inhibitor. With different concentration of quercetagetin and substrate, the absorbance changes were analyzed using Lineweaver-Burk plots (Figure 3B). The lines of quercetagetin intersected in the third quadrant and their slops increased with quercetagetin concentrations. The results indicated that quercetagetin inhibited tyrosinase in a mixed-type, meaning that quercetagetin bound to tyrosinase and tyrosinase-substrate complex to inhibit its activity. Similar phenomena were obtained in inhibition type of indole-carbohydrazides (Iraji et al., 2022).

**FIGURE 4:**
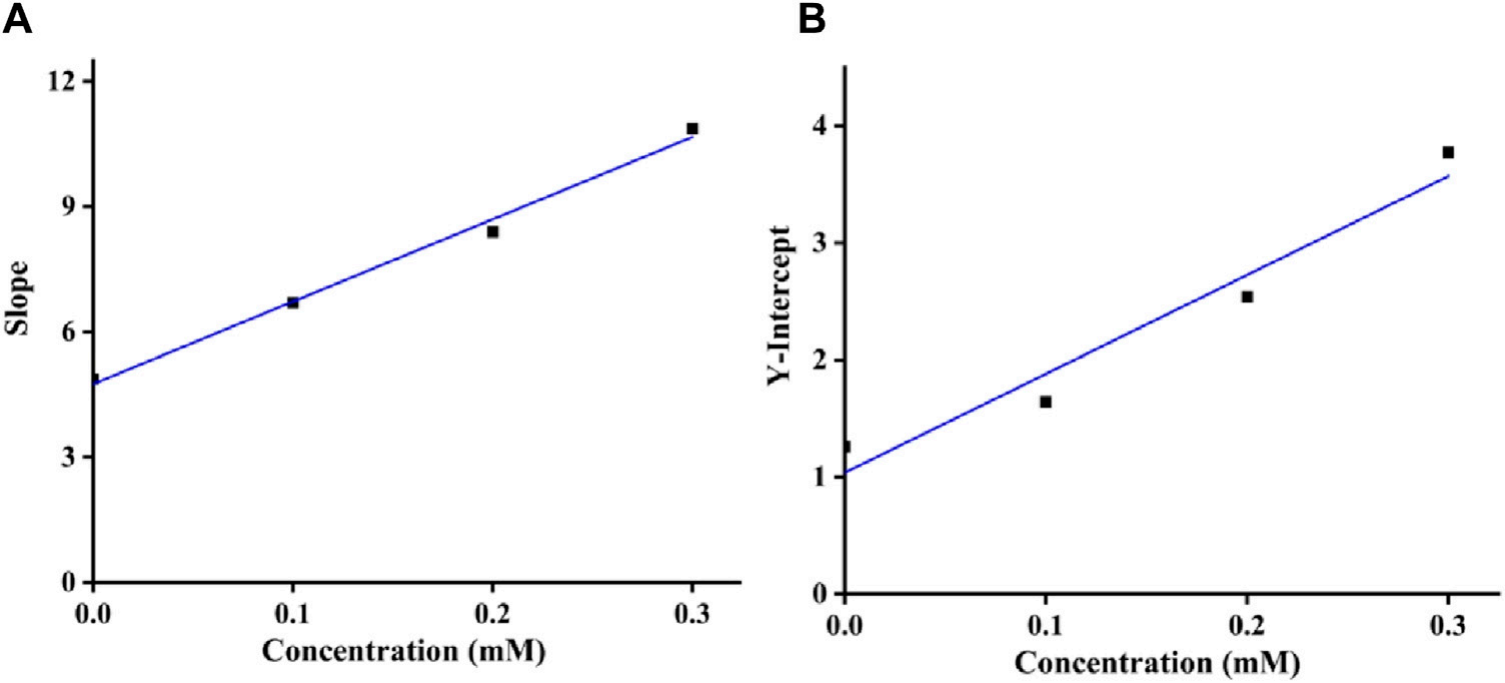
**(A)** *K*_i_ assay of quercetagetin; **(B)** *K*_is_ assay of quercetagetin.

Moreover, as shown in Figures 4A, B, the inhibition constants *K*_i_ and *K*_is_ were obtained from the secondary curves of inhibitory kinetics to be 0.24 and 0.12 mM. Smaller *K*_is_ value than *K*_i_ meant that binding force of quercetagetin with tyrosinase-substrate complex was stronger than with tyrosinase. That was to say that quercetagetin preferred to bind with tyrosinase-substrate complex.

### 2.3 Fluorescence quenching

**FIGURE 5:**
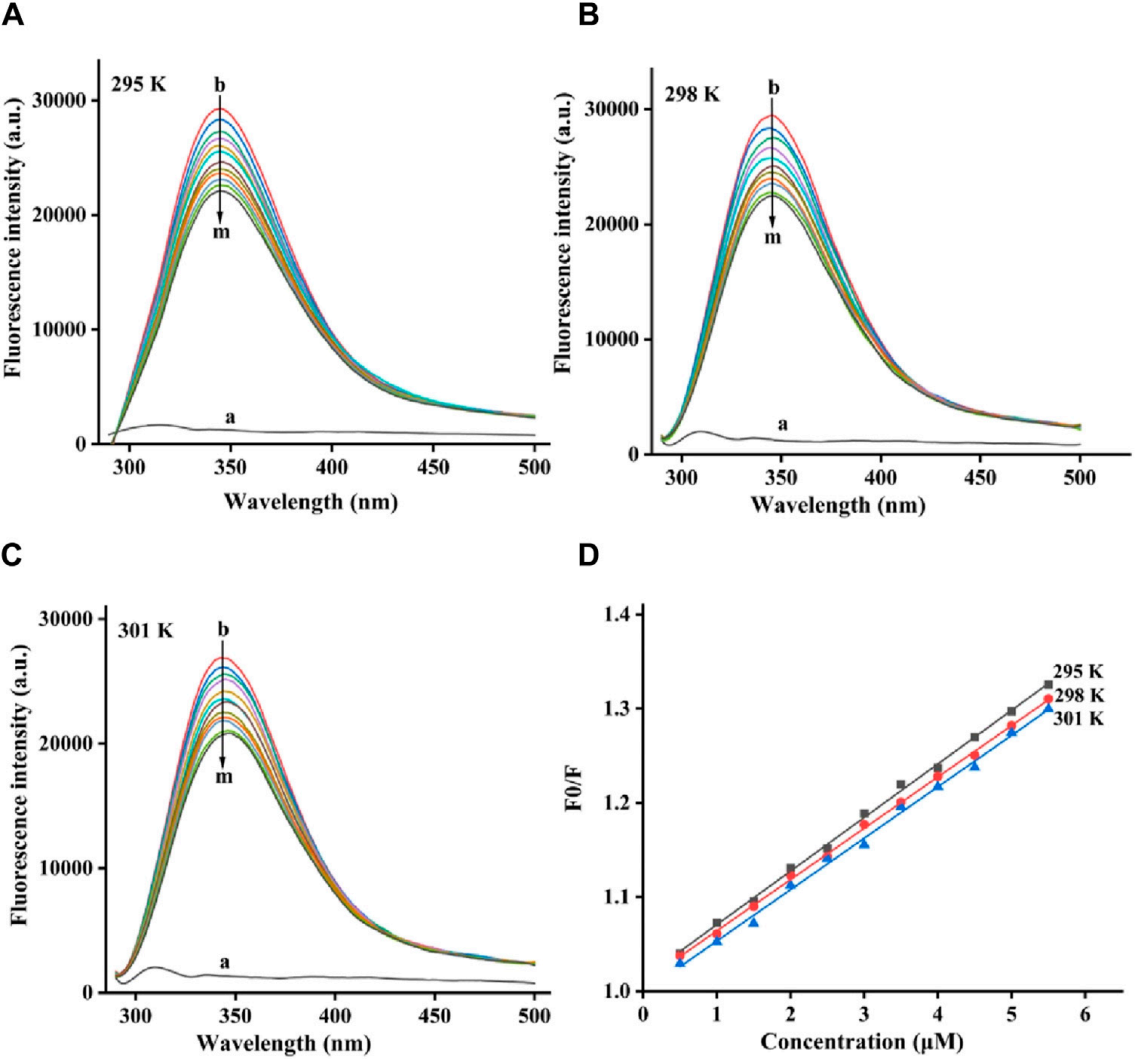
**(A–C)** Fluorescence quenching spectra of quercetagetin on tyrosinase at 295, 298, and 305 K, respectively; **(D)** Stern-Volmer plots of quercetagetin.

The fluorescence quenching process of tyrosinase by quercetagetin was investigated. For fluorescence spectra of tyrosinase, they all showed characteristic peaks at 344 nm at 295, 298, and 305 K, respectively (Figures 5A–C). But, quercetagetin did not show effective fluorescence spectra. Moreover, when treated by quercetagetin, tyrosinase presented the gradually decreasing peak intensity (Figures 5A–C), which indicated that quercetagetin bound to tyrosinase.

The fluorescence quenching data at 295, 298, and 305 K were future analyzed by Stern-Volmer plots. As is shown in Figure 5D the Stern-Volmer plots of quercetagetin, lines with different temperature presented good linearity, meaning that there was only one quenching type of static or dynamic in quenching process. Then the quenching constant (*K*_sv_) the bimolecular quench rate constant (*K*_q_) were obtained (Table 1). The *K*_sv_ results found that *K*_sv_ values decreased with the temperature. And *K*_q_ values at 295, 298, and 305 K were higher than 2 × 10^10^ L mol^−1^ S^−1^. Above results suggested the quenching process of quercetagetin against tyrosinase was a static process. This quenching type presented in the quenching of indole derivatives on α-glucosidase (Hu et al., 2024). The quenching process also was the process of thermodynamic changes. Thence, the thermodynamic parameters were calculated (Table 1). The negative ΔG value unlocked one spontaneous process of quercetagetin binding to tyrosinase. The positive ΔH and ΔS values suggested hydrophobic interactions as the important forces between quercetagetin and tyrosinase.

**TABLE 1:** Parameters of quercetagetin with tyrosinase.

T (K)	*K*_q_ (×10^12^ Lmol^−1^S^−1^)	*K*_sv_ (×10^4^Lmol^−1^)	△H (KJ/moL)	△G (KJ/moL)	△S (J/(mol·K)
295	5.69	5.69	25.34	−23.27	164.78
298	5.45	5.45		−23.76	
301	5.40	5.40		−24.26	

### 2.4 CD spectra

**FIGURE 6:**
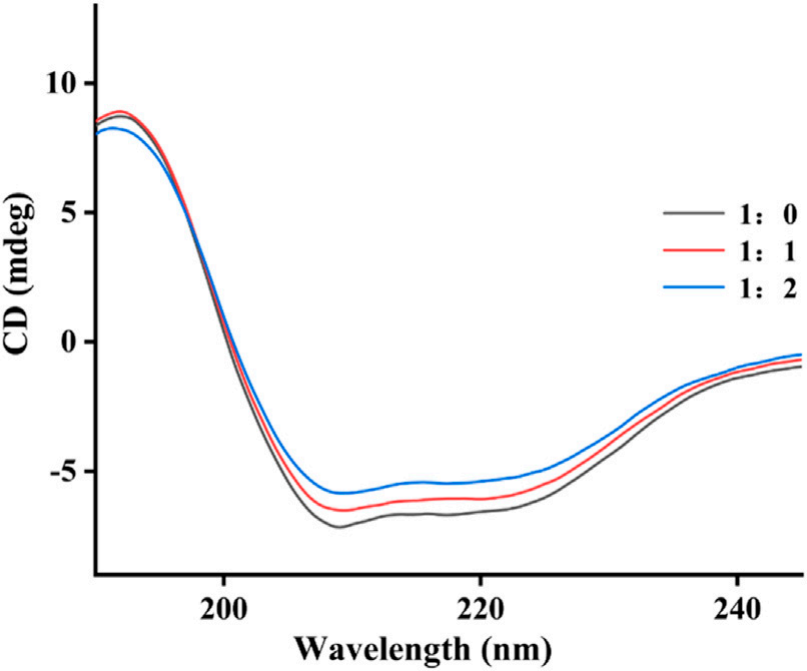
CD spectra of tyrosinase with quercetagetin.

The effect of quercetagetin on conformation change of tyrosinase was investigated using CD spectra. Figure 6 showed CD spectra of tyrosinase, the characteristic peaks around 210–220 nm stood for the peptide chains of tyrosinase. When treated with quercetagetin, the CD spectra of tyrosinase appeared some changes, meaning its conformation change. Then its secondary structure contents were calculated (Table 2) and found that quercetagetin treatment (molar ratio: 2:1) caused reduction of α-helix and random coils and increase of β-sheet and β-turn. These results indicated that quercetagetin treatment could lead to conformation changes of tyrosinase.

**TABLE 2:** The secondary structure contents of tyrosinase with quercetagetin.

Molar ratio	α-Helix (%)	β-Sheet (%)	β-Turn (%)	Random coils (%)
1:0	27.0	23.1	15.6	44.7
1:1	22.2	27.6	16.9	41.1
1:2	19.2	31.4	17.9	38.8

Note: Molar ratio means [tyrosinase]: [quercetagetin].

### 2.5 3D fluorescence spectra

**FIGURE 7:**
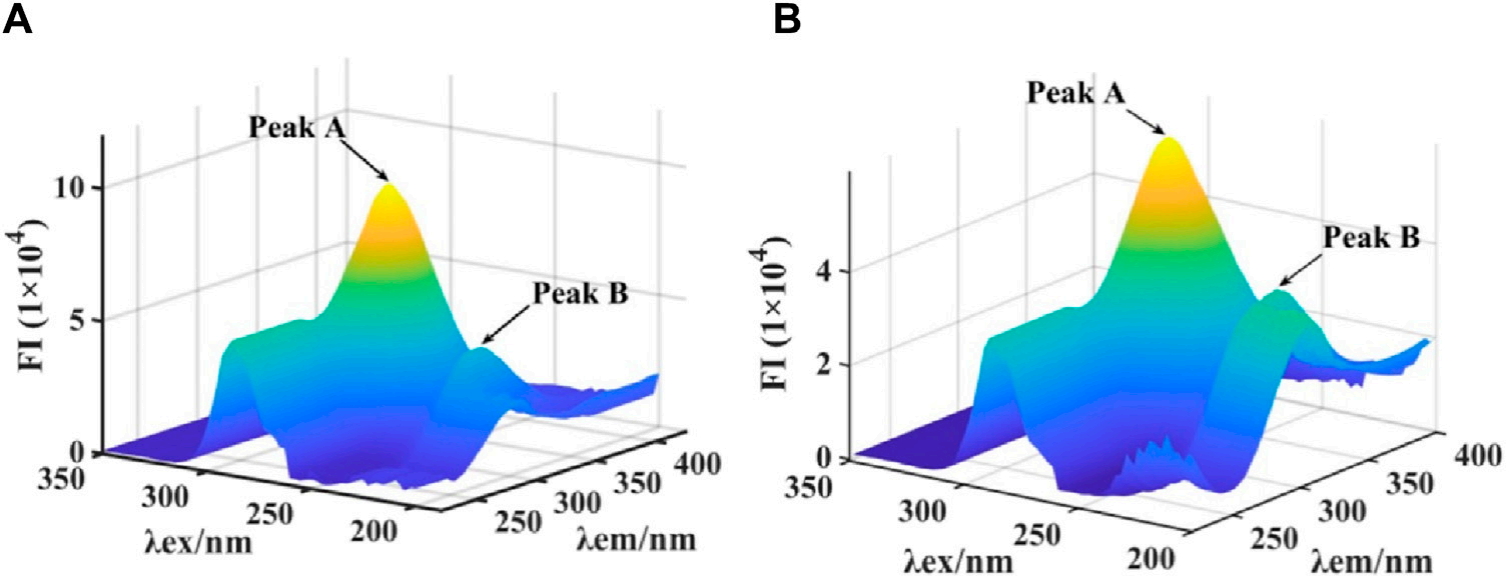
**(A,B)** 3D fluorescence of tyrosinase and quercetagetin-tyrosinase, respectively.

3D fluorescence spectra were monitored to further investigate the effect on conformation of tyrosinase by quercetagetin. As shown in Figure 7A the 3D fluorescence spectra tyrosinase, two characteristic peaks appeared, including Peak A for Tyr and Trp residues and Peak B for peptide backbone. When treated with quercetagetin, Peak A and Peak B were reduced the intensity by 35.4% and 13.3%, respectively (Figure 7B), which suggested that quercetagetin treatment would cause the changes of Tyr and Trp residues, and peptide backbone. That was to say that quercetagetin treatment would cause the conformation change of tyrosinase.

### 2.6 Molecular docking

**FIGURE 8:**
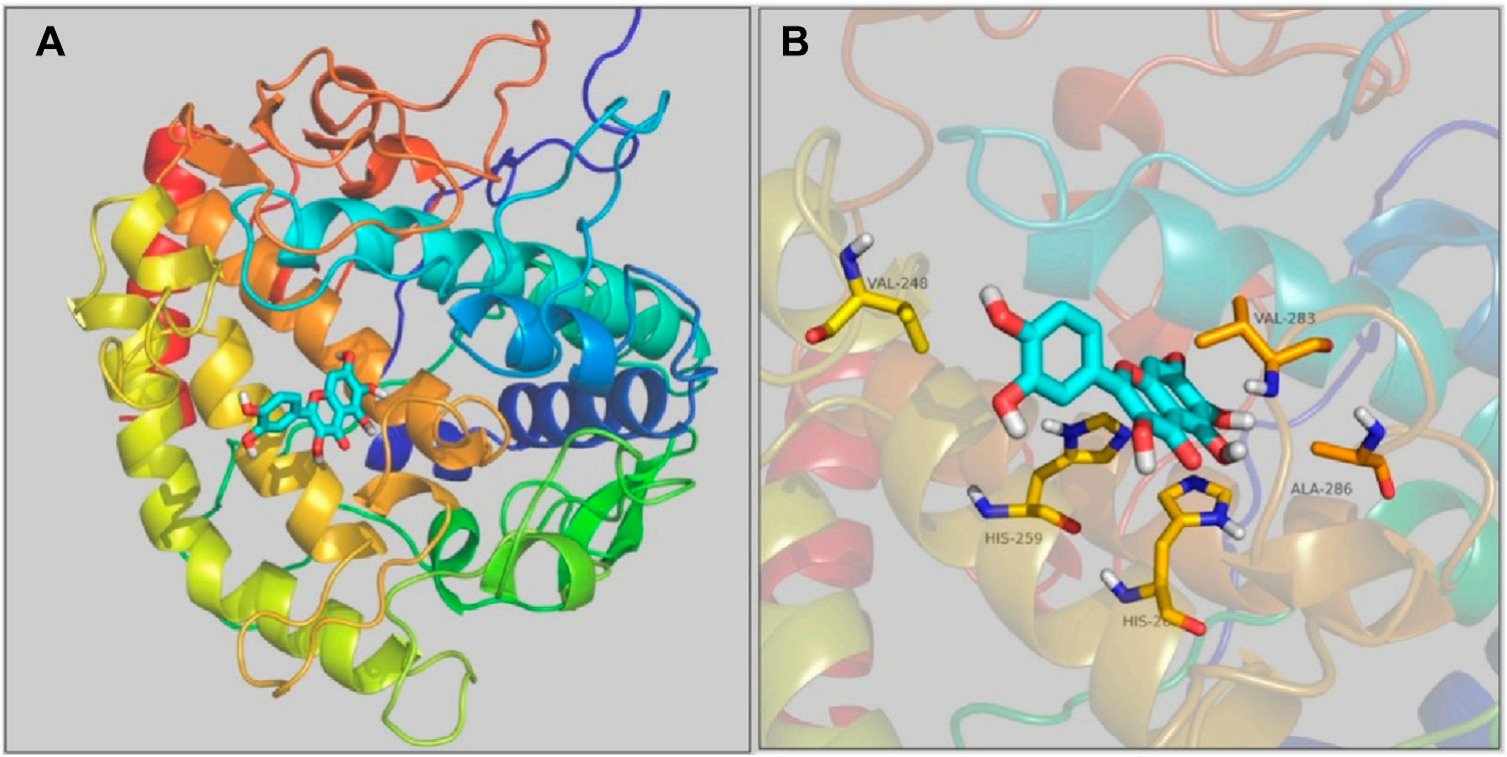
**(A,B)** The molecular docking of quercetagetin to tyrosinase.

The interaction of quercetagetin to tyrosinase was simulated by molecular docking method. As shown in Figure 8A the docking results, quercetagetin bound to the active pocket of tyrosinase with trihydroxychromone section in the active catalytic zone of active pocket. From Figure 8B the docking results in detail, it could be observed that quercetagetin made one hydrogen bond with His259 and hydrophobic interaction with Val248, His263, Val283, and Ala286. The results indicated that the interactions between quercetagetin and tyrosinase resulted in the reduction of tyrosinase activity.

### 2.7 Copper-chelating activity

**FIGURE 9:**
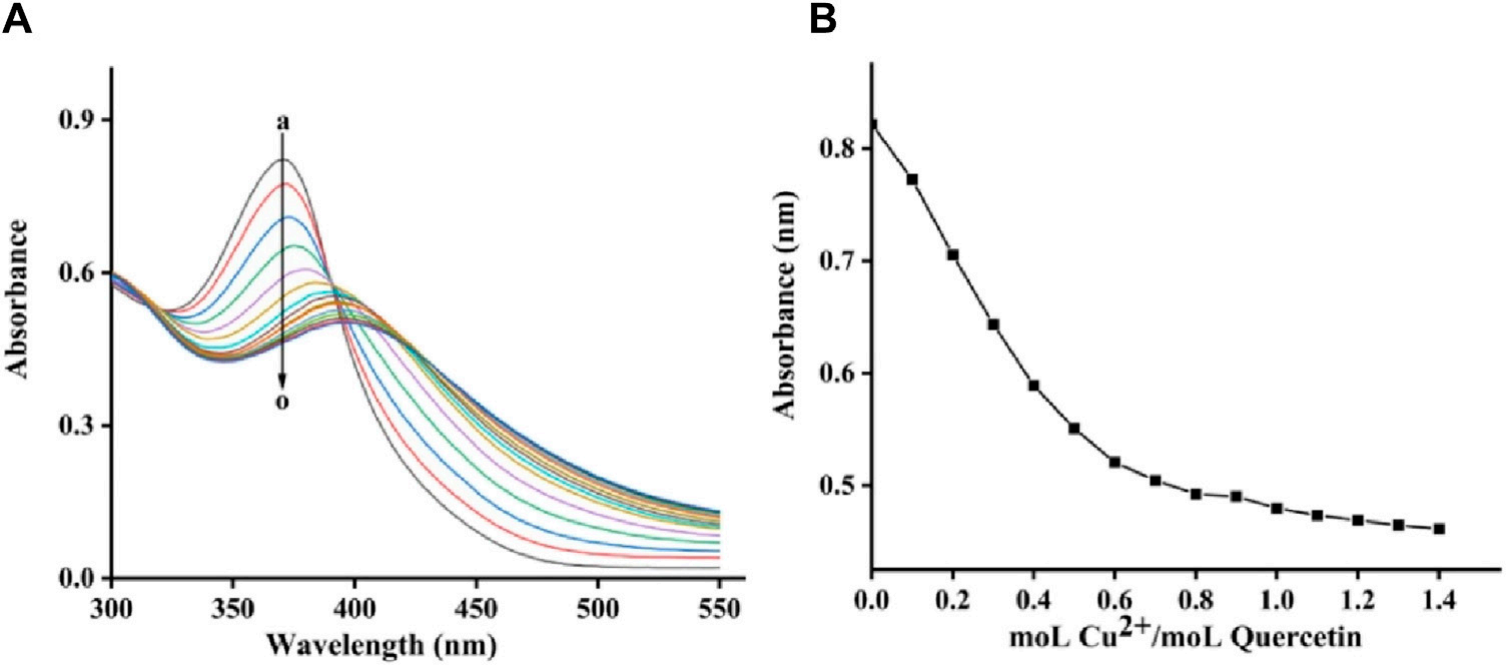
**(A)** UV spectra of quercetagetin-copper sulfate mixture; **(B)** UV peak of quercetagetin versus molar ratios of quercetagetin to copper sulfate.

The copper-chelating activity of quercetagetin was finally assayed using copper sulfate. Figure 9A showed the quercetagetin-copper sulfate mixture. Quercetagetin showed its UV spectra with characteristic peak at 370 nm. While, after added copper sulfate, the UV characteristic peak of quercetagetin was gradually decreased. These results indicated that copper might complex with quercetagetin to change its UV characteristic peak. The peak intensity at different molar ratios of quercetagetin to copper sulfate was analyzed (Figure 9B) and found that the decreasing trend of quercetagetin peak intensity become equilibrium, meaning that the binding molar ratio of quercetagetin with copper sulfate was 1. The copper-chelating activity of quercetagetin also might be one reason of the reduction of tyrosinase activity.

## 3 Conclusion

Tyrosinase is one important rate limiting enzyme in melanin synthesis, directly affecting the melanin synthesis. Quercetagetin is one active ingredient from marigold. In this study, the inhibition effects of quercetagetin against tyrosinase were investigated. The results showed that quercetagetin could inhibit the tyrosinase activity with IC_50_ value of 0.19 ± 0.01 mM and the inhibition type was a reversible mixed-type. Results of fluorescence quenching showed that quercetagetin could quench tyrosinase fluorescence in static process. CD and 3D fluorescence results showed interaction of quercetagetin to tyrosinase could change tyrosinase conformation to inhibit activity. Moreover, docking revealed details of quercetagetin’s interactions with tyrosinase.

## 4 Materials and methods

### 4.1 Tyrosinase activity assay

Tyrosinase inhibitory activity of quercetagetin was measured (Lu et al., 2023b). 10 μL of quercetagetin in DMSO solutions was added into 140 μL of tyrosinase in PBS solution and incubated for 10 min. 50 μL of L-dopa (in PBS) was added as the substrates. Then, the absorbance was measured at 475 nm on a microplate reader. Kojic acid was selected as the positive control. All experiments were tested in triplicate. Inhibition rate (%) = [(OD_1_-OD_0_)/OD_0_]×100%. OD_0_ and OD_1_ were the absorbance of tyrosinase and tyrosinase-quercetagetin mixture.

### 4.2 Inhibitory kinetics

The reversibility of quercetagetin against tyrosinase was analyzed at different concentration of quercetagetin with tyrosinase or L-dopa. And the inhibitory kinetics of quercetagetin against tyrosinase was analyzed at different concentration of quercetagetin with tyrosinase or L-dopa. The inhibition constants *K*_i_ and *K*_is_ were obtained from the secondary curves of inhibitory kinetics (Xu et al., 2020; Deng et al., 2022; Lin et al., 2023).

### 4.3 Fluorescence quenching

To the 3.0 mL of tyrosinase solution, 1 μL of quercetagetin was added by titration method (Li et al., 2024a; Min et al., 2024). The mixture was measured fluorescence spectra at excitation of 280 nm. The experiments were conducted at temperatures of 295, 298, and 305 K, respectively. The Stern-Volmer equation and the vanʼt Hoff equation were employed to obtained parameters.

### 4.4 CD spectra

To the 100 μL of tyrosinase solution, 1 μL of quercetagetin was added. CD spectra were measured at room temperature (Xiao et al., 2023; Li et al., 2024b). Tyrosinase solution without quercetagetin was also measured its CD spectra.

### 4.5 3D fluorescence spectra

To the 3.0 mL of tyrosinase solution, 1 μL of quercetagetin was added. 3D fluorescence spectra were measured (Wu X. et al., 2023).

### 4.6 Copper-chelating activity

To the 2 mL of quercetagetin solution, 5 μL of copper sulfate solution was gradually added. UV absorption spectra were detected. The molar ratios of quercetagetin to copper sulfate ranged from 10: 0 to 10: 14.

### 4.7 Molecular docking

Docking of quercetagetin with tyrosinase was simulated using the SYBYL software (Zhang et al., 2022; Feng et al., 2024). The tyrosinase crystal structure (PDB: 2Y9X) was optimized by removing water, adding hydrogen, and generation of active pocket. Quercetagetin structure was performed energy minimization. Thence, docking procedure was run in the default format.

## Data Availability

The raw data supporting the conclusion of this article will be made available by the authors, without undue reservation.
